# Predictive Value of Machine Learning Models for Cerebral Edema Risk in Stroke Patients: A Meta‐Analysis

**DOI:** 10.1002/brb3.70198

**Published:** 2025-01-08

**Authors:** Qi Deng, Yu Yang, Hongyu Bai, Fei Li, Wenluo Zhang, Rong He, Yuming Li

**Affiliations:** ^1^ Department of Neurology Tianjin Kanghui Hospital Tianjin China; ^2^ Department of Respiratory Tianjin Kanghui Hospital Tianjin China; ^3^ Department of General Surgery Tianjin Kanghui Hospital Tianjin China; ^4^ Department of Neurology PKUCare Rehabilitation Hospital Beijing China; ^5^ Department of Neurology Tianjin Kanghui Hospital Tianjin China

**Keywords:** cerebral edema, machine learning, predictive modeling, stroke

## Abstract

**Introduction:**

Stroke patients are at high risk of developing cerebral edema, which can have severe consequences. However, there are currently few effective tools for early identification or prediction of this risk. As machine learning (ML) is increasingly used in clinical practice, its effectiveness in predicting cerebral edema risk in stroke patients has been explored. Nonetheless, the lack of systematic evidence on its predictive value challenges the update of simple and user‐friendly risk assessment tools. Therefore, we conducted a systematic review to evaluate the predictive utility of ML for cerebral edema in stroke patients.

**Methods:**

We searched PubMed, Embase, Web of Science, and the Cochrane Database up to February 21, 2024. The risk of bias in selected studies was assessed using a bias assessment tool for predictive models. Meta‐analysis synthesized results from validation sets.

**Results:**

We included 22 studies with 25,096 stroke patients and 25 models, which were constructed using common and interpretable clinical features. In the validation cohort, the models achieved a concordance index (c‐index) of 0.840 (95% CI: 0.810–0.871) for predicting poststroke cerebral edema, with a sensitivity of 0.76 (95% CI: 0.72–0.79) and a specificity of 0.87 (95% CI: 0.83–0.90).

**Conclusion:**

ML models are significant in predicting poststroke cerebral edema, providing clinicians with a powerful prognostic tool. However, radiomics‐based research was not included. We anticipate advancements in radiomics research to enhance the predictive power of ML for poststroke cerebral edema.

## Graphical abstract

ML models can significantly predict cerebral edema following stroke. ML models can be integrated into electronic health record systems, thus providing real‐time support for clinical decision‐making.

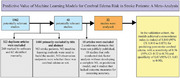

## 1 Introduction

Stroke is globally the second most prevalent cause of death and the third contributor to both mortality and disability (Potter, Tannous, and Vahidy 2022; Kuriakose and Xiao 2020; Mosconi and Paciaroni 2022). Around 15 million people worldwide experience stroke annually, and nearly 6 million people die of it (Asgedom et al. 2020). One in every six individuals is at risk of a stroke (Asgedom et al. 2020). Moreover, strokes have a significant economic impact. In high‐income countries, over 4% of total healthcare expenditures are attributed to the direct medical costs of strokes (Asgedom et al. 2020). Despite advancements in treatments such as intravenous thrombolysis and endovascular therapy that have significantly enhanced outcomes by preserving the ischemic penumbra, stroke complications—including cerebral edema, aspiration pneumonia, hemorrhagic transformation, depression, dementia, epilepsy, and cardiovascular complications—profoundly affect patient prognoses (Kuriakose and Xiao 2020; Asgedom et al. 2020; Hofmeijer et al. 2009; Chen et al. 2021; Hua, Liu, and Wu 2023; King et al. 2018). Particularly, cerebral edema can increase intracranial pressure, leading to hypoperfusion of brain tissues and insufficient oxygen supply, and ultimately severe neurological dysfunction and even death (Muscari et al. 2019).

Malignant cerebral edema (MCE) represents a severe post‐stroke complication, characterized by significant brain swelling within 1–3 days poststroke, leading to declined consciousness and displacement of midline brain structures (Q. M. Jiang, Yu, et al. 2022; Gu et al. 2022). Adverse outcomes of MCE encompass increased intracranial pressure, herniation, ischemic necrosis of brain tissue, and fatality (Miao et al. 2020). MCE occurs in 10%–30% of patients with hemispheric strokes and contributes to half of all in‐hospital deaths (Dhar 2020). The mortality rate of MCE can be as high as 78% (Hofmeijer et al. 2009).

MCE can be somewhat prevented through intracranial pressure monitoring and management, dehydration therapy, fluid intake restriction, hypertonic solutions, maintaining head elevation, ensuring adequate cerebral perfusion pressure, and controlling cerebral metabolism and hemodynamics to reduce intracranial pressure and brain tissue edema (Chen et al. 2021; Magid‐Bernstein et al. 2022). Early prediction of the risk of cerebral edema and performing decompressive hemicraniectomy (DHC) within 48 h before clinical deterioration can significantly reduce mortality and promote functional recovery (Hua, Liu, and Wu 2023; Foroushani et al. 2022). Therefore, accurate prediction of MCE is crucial for stroke management. In current clinical practice, predictions are usually based on clinical indicators (age, headache, vomiting, reduced consciousness, and National Institutes of Health Stroke Scale [NIHSS] score at admission) and imaging findings (the size of hypodense areas in cerebral parenchyma on head CT and lesion volume on brain MRI [magnetic resonance imaging]) (Hua, Liu, and Wu 2023; Q. M. Jiang, Yu, et al. 2022; Miao et al. 2020), while imaging indicators include. However, the predictive value of these methods is concerning.

With the extensive application of machine learning (ML) in the medical field, its predictive utility in the diagnosis and treatment of cerebral edema following strokes has been explored. However, there is still a lack of systematic evidence supporting its feasibility. Thereby, this paper aims to analyze the effectiveness of ML in predicting cerebral edema, aiming to provide evidence‐based support for developing simple assessment tools.

## 2 Methods

### 2.1 Study Registration

This systematic review was registered in the PROSPERO (CRD42024527045).

### 2.2 Eligibility Criteria

The studies were enrolled if they (1) involved stroke patients; (2) were case‐control studies, cohort studies, and cross‐sectional studies; (3) fully constructed predictive models for post‐stroke cerebral edema; (4) were published in English.

Studies were excluded for (1) review, meta‐analysis, guideline, or expert opinion; (2) only factor analysis without a complete ML model; (3) lacking any of the following outcome measures for assessing the predictive accuracy of the ML model: ROC curve, c‐statistic, recall, precision, concordance index (c‐index), accuracy, sensitivity, specificity, confusion matrix, diagnostic fourfold table, F1 score, or calibration curve; (4) sample size of less than 20 cases.

### 2.3 Search Strategy

PubMed, Web of Science, Embase, and Cochrane databases were comprehensively searched from their inception to February 21, 2024 using a combination of keywords and subject headings, and the language was restricted to English. The specific search strategy is presented in Table S1.

### 2.4 Study Selection and Data Extraction

The retrieved literature was imported into EndNote. After duplicates were excluded, the titles or abstracts were read to preliminarily identify eligible studies. Full texts were then assessed in detail to decide the final inclusion.

Before data extraction, a standardized data extraction spreadsheet was established, which included title, first author, publication year, author's country, study type, patient source, stroke type, treatment background, diagnostic criteria for cerebral edema, follow‐up duration, number of cerebral edema cases, the total number of cases, the number of cerebral edema cases in the training set, total cases in the training set, generation method of validation sets, overfitting methods, the number of cerebral edema cases in the validation set, the number of cases in the validation set, handling method of missing value, variable screening/feature selection method, model type, and modeling variables.

Literature screening and information extraction were independently undertaken by two researchers (D.Q. and Y.Y.), and any disputes were addressed by a third researcher (L.Y.M.).

### 2.5 Risk of Bias in Studies

The risk of bias in enrolled articles was judged using PROBAST (de Jong et al. 2021). PROBAST comprised numerous questions across participants, predictors, outcomes, and statistical analysis. The four domains included two, three, six, and nine specific questions, respectively, each with three possible answers (yes/probably yes, no/probably no, and no information). The domain was deemed high risk if at least one question was answered as no/probably no. The domain was deemed low risk if all questions were answered as yes/probably yes.

In assessing the risk of bias among the studies included, we encountered several challenges, particularly with respect to the predictors, outcomes, and statistical analyses. Regarding the predictors, some studies were rated as “unclear” due to insufficient information to determine whether the assessors evaluated the predictors in a blinded manner. Specifically, the independence and objectivity of the predictor evaluation cannot be accurately judged because it was not explicitly detailed whether the results were known during the assessment process.

Similarly, in terms of outcomes, certain studies were deemed “unclear” concerning whether predictors were known or utilized in determining outcomes. This was attributed to inadequate reporting on whether and how predictors were considered during the process of outcome determination. This uncertainty complicates the interpretation of results and may undermine the reliability of conclusions.

With regard to statistical analyses, studies were also rated as “unclear” owing to the absence of detailed reports on the management of missing data, omitted predictors, or outcomes. This situation typically arose from the lack of specific details on how missing data were addressed, including whether appropriate statistical techniques were employed to minimize bias risks. Furthermore, there was a lack of transparency in addressing data complexity, such as considering potential confounders or interactions. These issues hindered a comprehensive assessment of the statistical quality and robustness of the study results.

Two researchers (D.Q. and Y.Y.) independently undertook the risk of bias assessment based on PROBAST and cross‐checked upon completion. Any disputes were addressed by a third researcher (L.Y.M.).

### 2.6 Outcomes

The primary outcomes of interest were the c‐index, sensitivity, and specificity in ML predictions for post‐stroke cerebral edema. The secondary outcome is predictors.

### 2.7 Synthesis Methods

Meta‐analysis of c‐index was conducted to evaluate the overall accuracy of ML models. For original studies where the 95% CI and standard error of the c‐index were missing, the standard error was estimated based on the methodology outlined by Debray et al. (2019). When heterogeneity (*I* ^2^) > 50%, a random‐effects model was utilized for meta‐analysis.

Additionally, meta‐analyses of sensitivity and specificity were done using a bivariate mixed‐effects model based on the diagnostic fourfold table. Since most original studies did not provide the diagnostic 2 × 2 table, it was calculated using sensitivity, specificity, and case numbers. This meta‐analysis was done using R4.2.0.

## 3 Results

### 3.1 Study Selection

**FIGURE 1:**
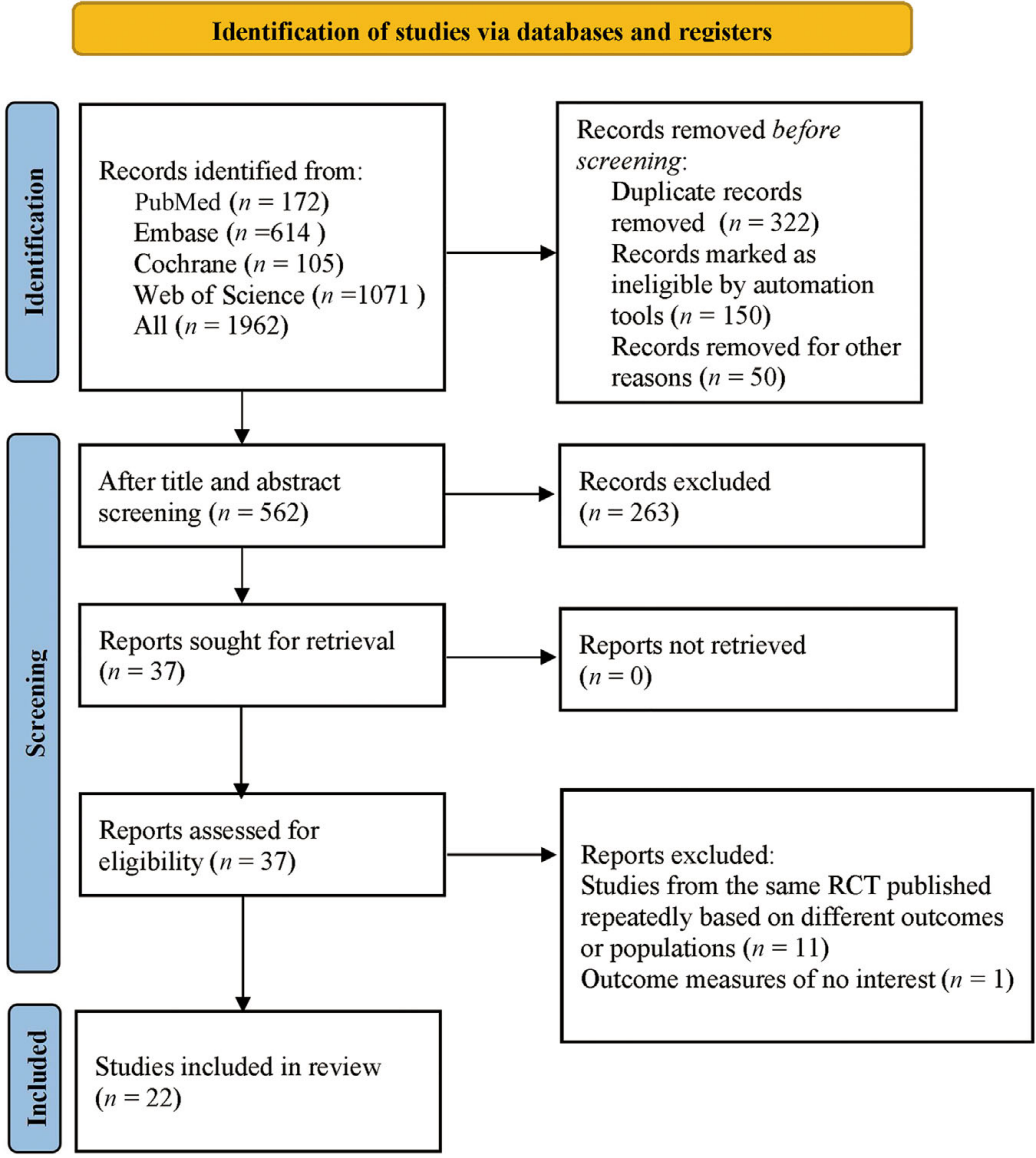
Flowchart of study selection process.

We retrieved 1962 articles from various databases. A total of 322 duplicate articles were excluded, with 260 marked by software and 62 identified manually. After screening the titles and abstracts, an additional 1600 articles were excluded. For the remaining 40 articles, the full texts were downloaded and thoroughly reviewed. Following the full‐text review, 18 articles were excluded, with 2 conference abstracts that were not publicly published, 10 studies that only conducted risk factor analysis without developing a complete ML or prediction model, and 6 studies that lacked outcome measures for assessing accuracy. As a result, 22 articles (Q. M. Jiang, Yu, et al. 2022; Foroushani et al. 2022; Zhang et al. 2018; Zeng et al. 2022; Yoo et al. 2013; Xie et al. 2023; Wen et al. 2023; Mohammadian Foroushani et al. 2021; Kumar et al. 2022; L. Jiang, Zhang, et al. 2022; L. Jiang et al. 2023; Hoffman et al. 2023; Fu et al. 2020; Foroushani et al. 2020; Dhar et al. 2020; Dhar et al. 2018; Chen et al. 2016; Bustamante et al. 2017; Wu et al. 2023; Pu et al. 2023; Du et al. 2020; Meinel et al. 2022) were included (Figure 1).

### 3.2 Study Characteristics

A total of 22 included papers were published between 2013 and 2023 and mainly conducted in China (Q. M. Jiang, Yu, et al. 2022; Zhang et al. 2018; Zeng et al. 2022; Xie et al. 2023; Wen et al. 2023; L. Jiang, Zhang, et al. 2022; L. Jiang et al. 2023; Fu et al. 2020; Wu et al. 2023; Pu et al. 2023; Du et al. 2020), the United States (Foroushani et al. 2022; Yoo et al. 2013; Mohammadian Foroushani et al. 2021; Kumar et al. 2022; Hoffman et al. 2023; Foroushani et al. 2020; Dhar et al. 2020; Dhar et al. 2018; Chen et al. 2016), Spain (Chen et al. 2016; Bustamante et al. 2017), and Switzerland (Meinel et al. 2022), with China being the primary initiator. The research design consisted of 11 retrospective cohort studies (Q. M. Jiang, Yu, et al. 2022; Foroushani et al. 2022; Wen et al. 2023; Kumar et al. 2022; L. Jiang, Zhang, et al. 2022; L. Jiang et al. 2023; Hoffman et al. 2023; Fu et al. 2020; Chen et al. 2016; Du et al. 2020; Meinel et al. 2022) and 11 prospective cohort studies (Zhang et al. 2018; Zeng et al. 2022; Yoo et al. 2013; Xie et al. 2023; Mohammadian Foroushani et al. 2021; Foroushani et al. 2020; Dhar et al. 2020; Dhar et al. 2018; Bustamante et al. 2017; Wu et al. 2023; Pu et al. 2023). Most studies (15) were single‐center (Q. M. Jiang, Yu, et al. 2022; Zhang et al. 2018; Zeng et al. 2022; Yoo et al. 2013; Xie et al. 2023; Wen et al. 2023; Kumar et al. 2022; L. Jiang, Zhang, et al. 2022; L. Jiang et al. 2023; Hoffman et al. 2023; Fu et al. 2020; Dhar et al. 2018; Chen et al. 2016; Pu et al. 2023; Meinel et al. 2022), while seven were multicenter (Foroushani et al. 2022; Mohammadian Foroushani et al. 2021; Foroushani et al. 2020; Dhar et al. 2020; Bustamante et al. 2017; Wu et al. 2023; Du et al. 2020). The primary focus was ischemic stroke. Treatment backgrounds included antiplatelet therapy (L. Jiang et al. 2023), neuroprotective agents (Wu et al. 2023), rehabilitation therapy (Wu et al. 2023), dehydration treatment (Zhang et al. 2018) (intravenous injection of 20% mannitol or 10% hypertonic saline), intravenous thrombolysis (Yoo et al. 2013; L. Jiang et al. 2023), endovascular treatments (Q. M. Jiang, Yu, et al. 2022; Zeng et al. 2022; Wen et al. 2023; L. Jiang, Zhang, et al. 2022; L. Jiang et al. 2023; Hoffman et al. 2023; Fu et al. 2020; Wu et al. 2023; Pu et al. 2023; Du et al. 2020; Meinel et al. 2022) (endovascular thrombectomy and mechanical thrombectomy), and surgical interventions such as DHC (Foroushani et al. 2022). Diagnostic criteria for cerebral edema were based on clinical manifestations (Zhang et al. 2018) and imaging examinations (Zhang et al. 2018; Wen et al. 2023; Bustamante et al. 2017; Wu et al. 2023; Du et al. 2020), with clinical criteria including an increase in NIHSS score (Zeng et al. 2022; Xie et al. 2023; Pu et al. 2023) of ≥ 4 or an increase in the consciousness assessment part of the NIHSS score of ≥ 1, and imaging criteria including low‐density lesions covering > 50% of the middle cerebral artery territory (Zeng et al. 2022; Mohammadian Foroushani et al. 2021; Hoffman et al. 2023), local signs of cerebral edema (Q. M. Jiang, Yu, et al. 2022; Foroushani et al. 2022; Zhang et al. 2018; Zeng et al. 2022; Xie et al. 2023; Wen et al. 2023; L. Jiang, Zhang, et al. 2022; L. Jiang et al. 2023; Hoffman et al. 2023; Bustamante et al. 2017; Wu et al. 2023; Pu et al. 2023; Du et al. 2020) (compression of lateral ventricles, disappearance of sulci, septal shift, pineal layer thickness > 5 mm, and occlusion of basal cisterns), and significant changes in ΔCSF (cerebrospinal fluid volume) (Foroushani et al. 2022; Kumar et al. 2022; Dhar et al. 2020; Chen et al. 2016). Follow‐up duration ranged from 18 h to 1 year. Validation methods primarily included internal random sampling methods, such as k‐fold cross‐validation (Foroushani et al. 2022; Zeng et al. 2022; Xie et al. 2023; Hoffman et al. 2023; Foroushani et al. 2020; Chen et al. 2016), Bootstrap (L. Jiang, Zhang, et al. 2022; L. Jiang et al. 2023; Du et al. 2020), leave‐one‐out cross‐validation (L. Jiang, Zhang, et al. 2022), stratified cross‐validation (Foroushani et al. 2022), random forests (Dhar et al. 2018; Bustamante et al. 2017), and k‐nearest neighbors imputation (Meinel et al. 2022). The study included 3 ANN models (Foroushani et al. 2022; L. Jiang, Zhang, et al. 2022; Hoffman et al. 2023), 4 support vector machine (SVM) models (Zeng et al. 2022; L. Jiang, Zhang, et al. 2022; Hoffman et al. 2023; Fu et al. 2020), 1 Adaboost model (L. Jiang, Zhang, et al. 2022), 1 GBM model (Zeng et al. 2022), 2 KNN models (Zeng et al. 2022; L. Jiang, Zhang, et al. 2022), 14 LR models (Q. M. Jiang, Yu, et al. 2022; Foroushani et al. 2022; Zhang et al. 2018; Zeng et al. 2022; Yoo et al. 2013; Xie et al. 2023; Wen et al. 2023; Fu et al. 2020; Foroushani et al. 2020; Dhar et al. 2020; Bustamante et al. 2017; Wu et al. 2023; Pu et al. 2023; Du et al. 2020), 1 NB model (L. Jiang, Zhang, et al. 2022), 6 RF models (Zeng et al. 2022; L. Jiang, Zhang, et al. 2022; Hoffman et al. 2023; Fu et al. 2020; Chen et al. 2016; Bustamante et al. 2017), and 2 XGBoost model (Zeng et al. 2022; Meinel et al. 2022). In summary, the literature included demonstrated the application of multidimensional data in predicting cerebral edema, as well as the diversity of models and validation methods. More baseline details are displayed in Table S2.

### 3.3 Risk of Bias in Studies

**FIGURE 2:**
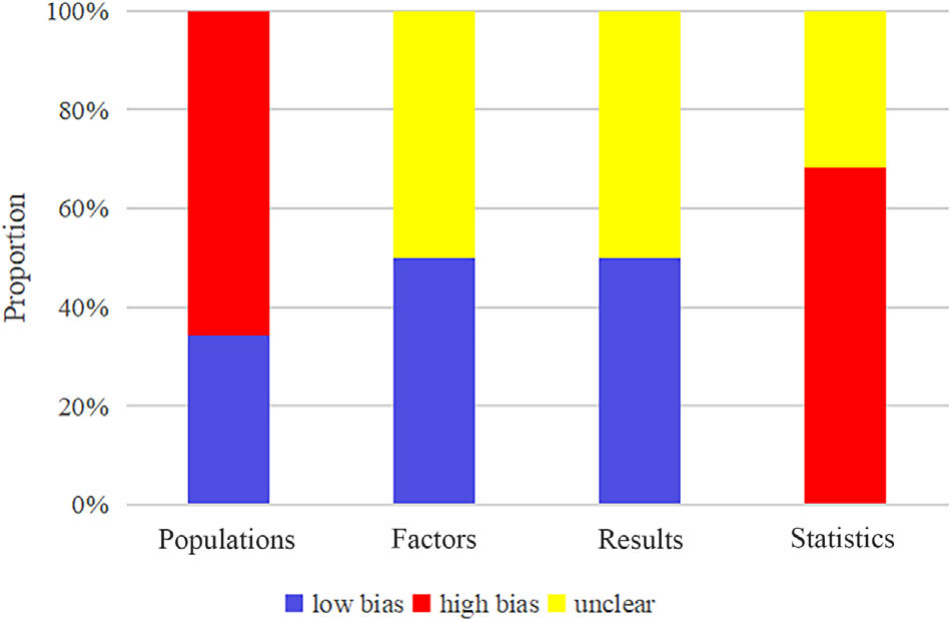
Risk of bias assessment results for included predictive models.

In all enrolled literature, 25 predictive models were derived from case‐control studies, potentially introducing a high risk of bias in the selection of study subjects. Additionally, in 19 models, it was unclear whether these predictive factors were assessed without knowledge of the outcome information, so these models were marked as unclear risk of bias. Similarly, for outcome assessment, in 19 models, it was uncertain whether cerebral edema was evaluated with knowledge of the distribution of predictive factors for cerebral edema, leading to an unclear risk of bias. In terms of statistical analysis, 26 models did not meet the requirement of EPV (events per variable) ≥ 20 or lacked an independent validation set, potentially introducing a high risk of bias. Additionally, one model was based on radiomics data, and due to the inability to calculate EPV, the risk of bias was also marked as unclear (Figure 2).

### 3.4 Predictors

In the 22 original studies, the predictive factors were categorized into seven major groups. The first group was demographic characteristics, including age and gender. The second group was clinical features, encompassing blood pressure, body temperature, pupil response, stroke location and type, NIHSS score, modified Rankin Scale (mRS) score, Glasgow Coma Scale (GCS) score, and APACHE II (Acute Physiology and Chronic Health Evaluation II) score. The third group was imaging features, including the disappearance of the basal cistern, low‐density areas, ASPECTS (Alberta Stroke Program Early CT Score), high‐signal areas in white matter, diffusion‐weighted imaging (DWI) lesion volume, middle cerebral artery (MCA) hyperdense vessel sign, collateral circulation score, multiple perfusion imaging parameters (*T*_max_ > 10 s, *T*_max_ > 6 s, cerebral blood flow < 30%), cerebral atrophy, ischemic lesion volume and core volume, reperfusion grade (mTICI, Modified Treatment in Cerebral Ischemia), and midline shift. The fourth group was treatment information, including ultra‐acute reperfusion therapy, intravenous thrombolysis, bridging therapy, and endovascular treatment. The fifth group was complications, including atrial fibrillation, history of hypertension, hyperlipidemia, diabetes, alcohol abuse, smoking, active cancer, prestroke disability, and respiratory infection. The sixth group was laboratory indicators, including fasting blood glucose levels, glycated hemoglobin levels, C‐reactive protein, creatinine, white blood cell count, neutrophil count, monocyte count, procalcitonin (PCT) level, neuron‐specific enolase (NSE) level, and blood calcium. The seventh group was time variables, including the time of onset, time of arrival at the hospital, and time of reperfusion (Table S2).

### 3.5 Meta‐Analysis

#### 3.5.1 Synthesized Results

**FIGURE 3:**
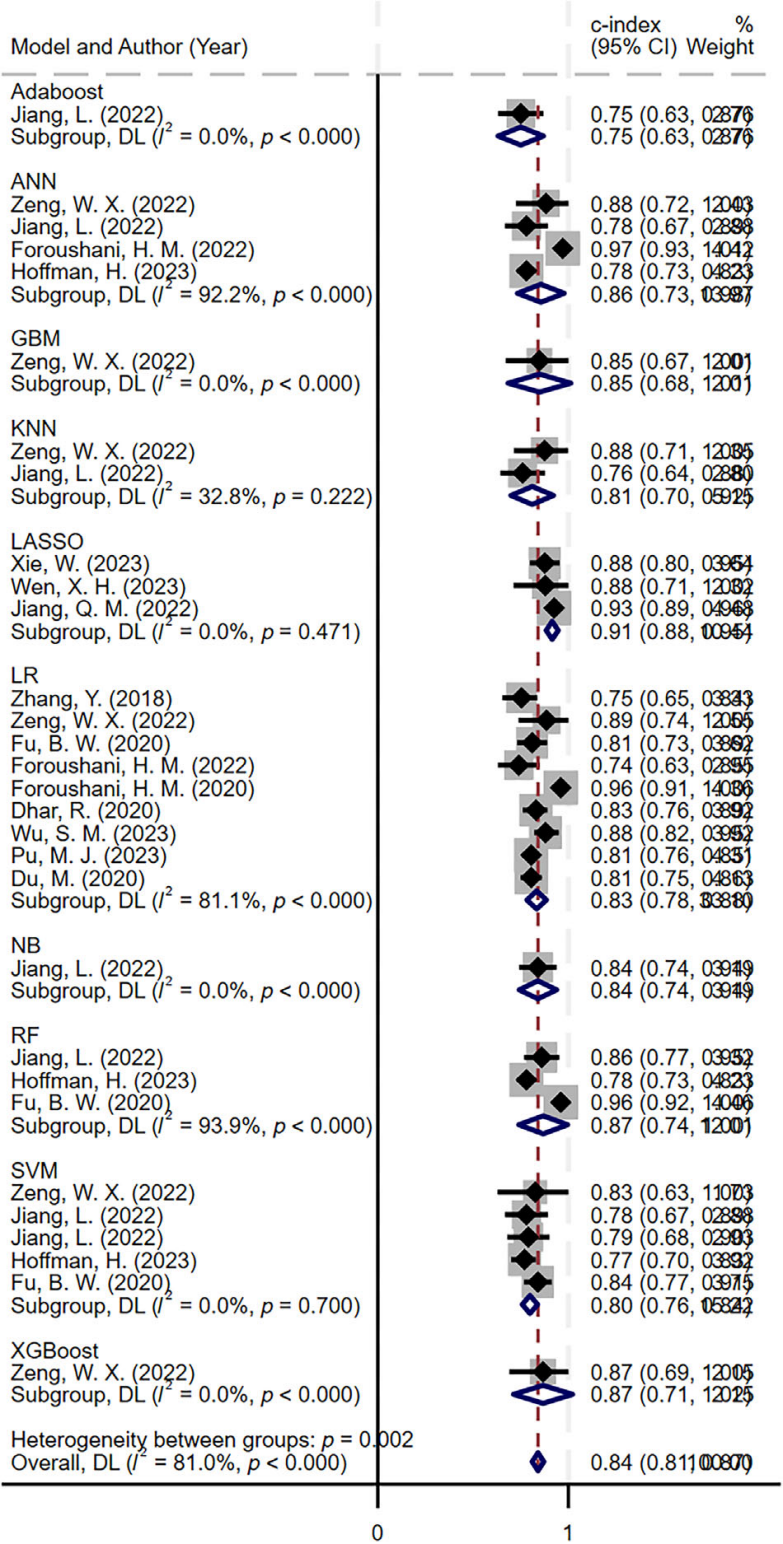
Forest plot of concordance index (c‐index) meta‐analysis for machine learning (ML) prediction of cerebral edema in validation sets. The pooled c‐index across the validation datasets indicates an overall predictive power of the models, with a c‐index of 0.840 (95% CI: 0.810–0.871).

**FIGURE 4:**
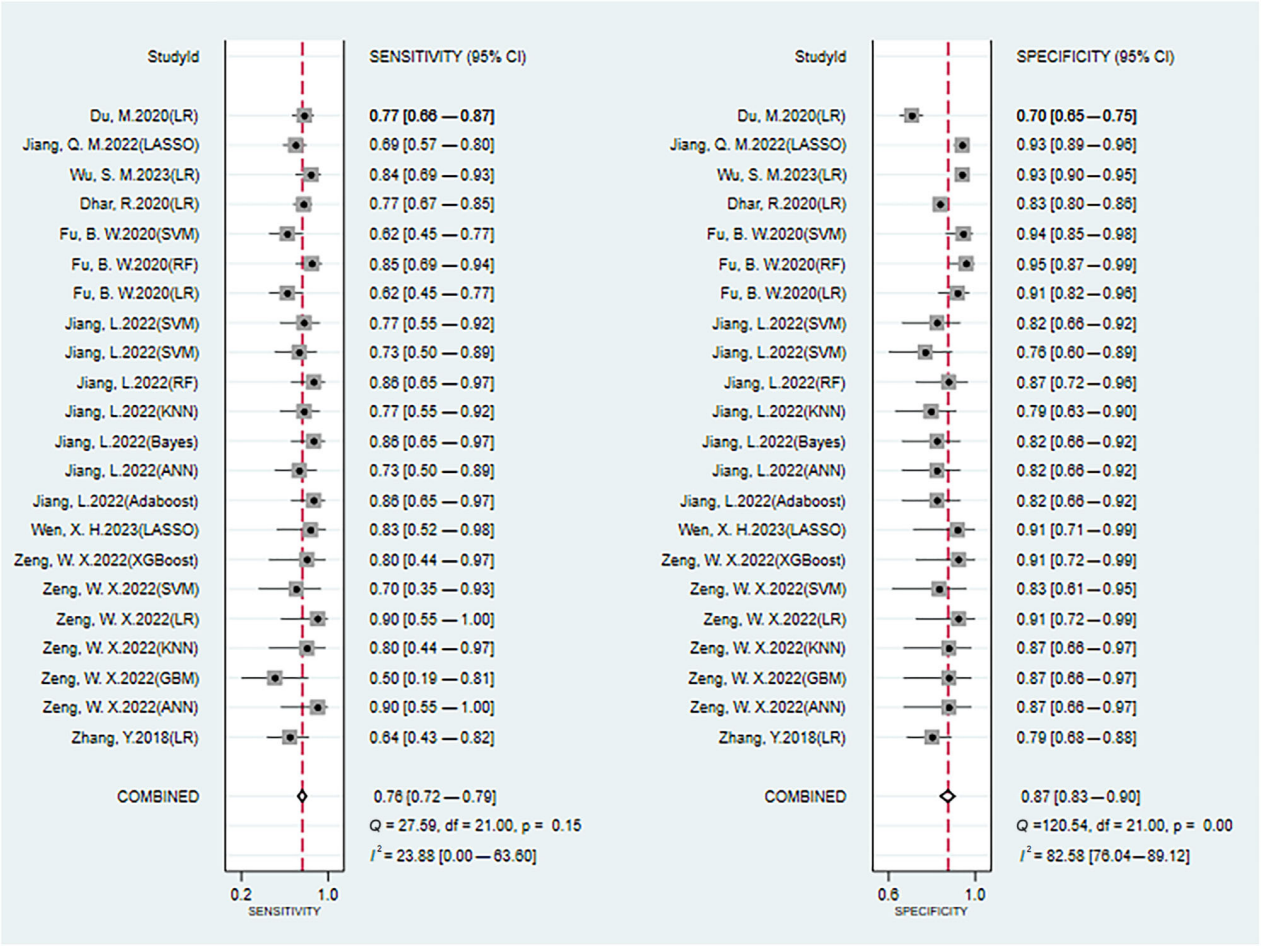
Forest plot of sensitivity and specificity meta‐analysis for ML prediction of cerebral edema in validation sets. The pooled sensitivity and specificity are 0.76 (95% CI: 0.72–0.79) and 0.87 (95% CI: 0.83–0.90), respectively, demonstrating the robustness of the ML prediction models in identifying true positive cases and minimizing false positives. CI, confidence interval; ML, machine learning.

A total of 30 different prediction models were included, each equipped with its independent validation dataset. To appraise the predictive power of these models, a random‐effects model was utilized. The meta‐analysis indicated that, across the validation datasets, the models achieved a pooled c‐index of 0.840 (95% CI: 0.810–0.871). Furthermore, the pooled sensitivity and specificity were 0.76 (95% CI: 0.72–0.79) and 0.87 (95% CI: 0.83–0.90), respectively, providing quantitative evidence of model validity (Figures 3 and 4).

#### 3.5.2 Subgroup Analysis

Given the potential variations in predictive performance across different models, this study further conducted subgroup analyses to explore the independent predictive value of various types of models. Logistic regression, SVM, and neural networks were more frequently utilized among the models studied. Specifically, the c‐index for logistic regression models was 0.833 (95% CI: 0.782–0.885), while for SVM and neural network models, the c‐index was 0.798 (95% CI: 0.758–0.838) and 0.855 (95% CI: 0.733–0.978), respectively. In terms of sensitivity, the values for logistic regression, SVM, and neural network models were 0.75 (95% CI: 0.69–0.81), 0.70 (95% CI: 0.58–0.80), and 0.74–0.90, respectively. Specificity values were 0.86 (95% CI: 0.78–0.91), 0.85 (95% CI: 0.76–0.91), and 0.81–0.87, respectively (Table S3).

### 3.6 Sensitivity Analysis and Reporting Biases

**FIGURE 5:**
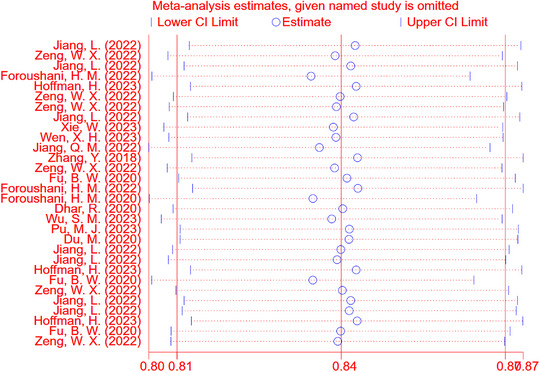
Sensitivity analysis forest plot of c‐index meta‐analysis for ML prediction of cerebral edema in validation sets. The sensitivity analysis demonstrates the robustness of our meta‐analysis, with consistent results across different models included in the study. This plot highlights the stability and reliability of the pooled c‐index value, indicating that the findings are not significantly influenced by individual studies.

**FIGURE 6:**
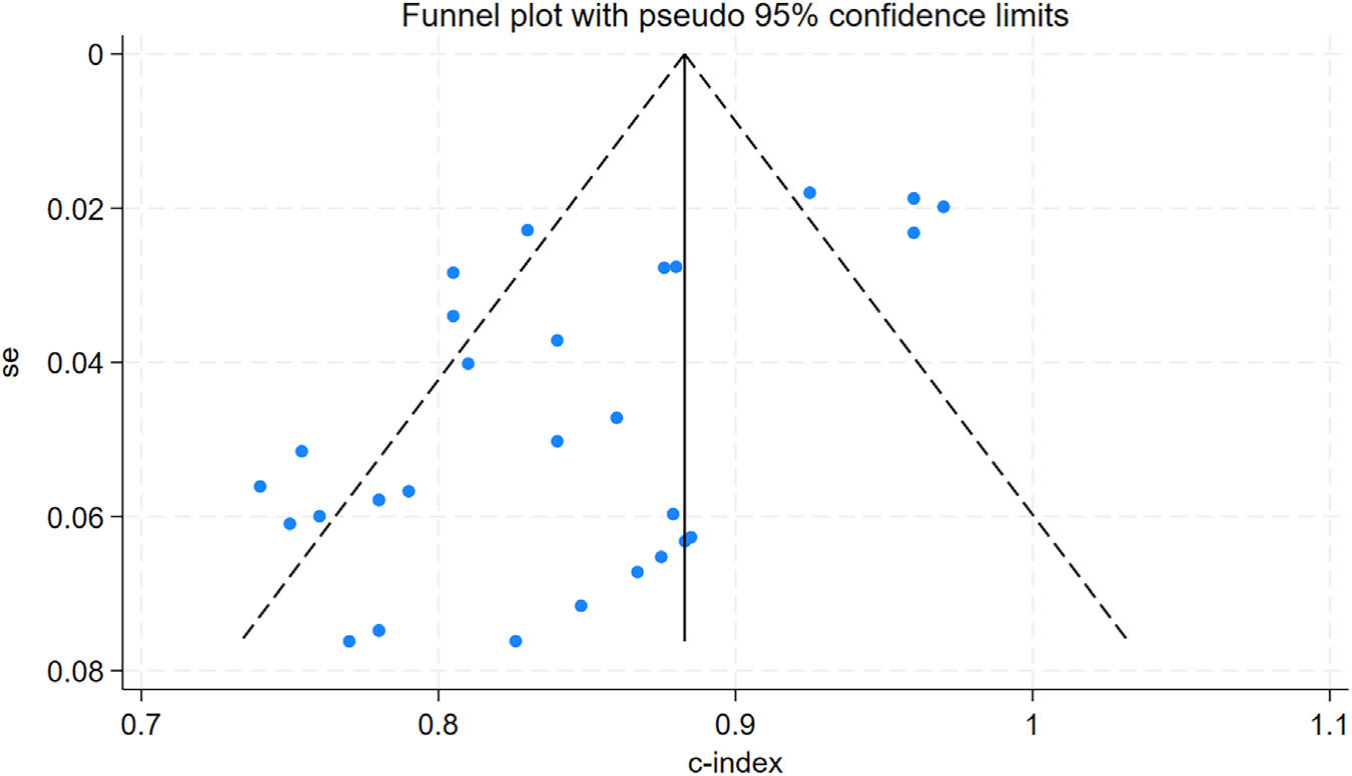
Funnel plot of sensitivity and specificity meta‐analysis for ML prediction of cerebral edema in validation sets. The funnel plot illustrates potential publication bias, with a significant deviation observed (*p* < 0.05). This suggests that smaller studies with less favorable outcomes may be underrepresented in the literature, potentially affecting the overall interpretation of the meta‐analysis results.

Sensitivity analysis of c‐index indicated that our meta‐analysis had desirable robustness. The funnel plot suggested significant publication bias (*p* < 0.05) (Figures 5 and 6).

## 4 Discussion

### 4.1 Summary of the Main Findings

In our systematic review, we included 22 studies, encompassing 25 ML models based on common clinical features. None of these studies reported models built using radiomics features. The meta‐analysis results demonstrated that these models exhibited good accuracy in predicting poststroke cerebral edema. Specifically, in the validation set, the pooled c‐index reached 0.840 (95% CI: 0.810–0.871), with 0.76 sensitivity (95% CI: 0.72–0.79). The logistic regression models, SVM models, and neural network models were primarily utilized, with c‐indices of 0.833 (95% CI: 0.782–0.885), 0.798 (95% CI: 0.758–0.838), and 0.855 (95% CI: 0.733–0.978), respectively.

### 4.2 Comparison With Other Reviews

The research and development of tools for forecasting cerebral edema in stroke patients have received significant attention recently. T Kasner score, EDEMA score, DASH score, MBE score, TURN score, and E‐score have been proposed and implemented in clinical practice (Foroushani et al. 2022; Tang et al. 2024).

A systematic review indicates that Kasner score, EDEMA score, DASH score, and MBE score exhibit notable capability in predicting MCE, each with a c‐index of 0.70 or higher (Wu et al. 2018). In contrast, the TURN score and E‐score are not comprehensively evaluated. Our results reveal that ML‐based prediction models achieve an overall c‐index of 0.840 (95% CI: 0.810–0.871), significantly surpassing the performance of traditional tools. This suggests broader applications for these ML models in clinical practice. Therefore, we should consider updating these predictive tools in future research.

A recent meta‐analysis (Miao et al. 2020) evaluated predictors of poststroke cerebral edema, including ASPECTS score, platelet count, DWI infarct volume, GCS score at admission, stroke history, mean systolic blood pressure, proximal ICA occlusion, NIHSS score at admission, admission temperature, age, large artery atherosclerosis, atrial fibrillation, cardioembolic stroke, blood glucose level, history of diabetes, hypertension, sex, smoking history, clinical symptom severity, infarct volume, extent of involved infarct vessels, blood pressure, and white blood cell count. In contrast, our study employed an ML model that integrated additional key features such as disappearance of the basal cistern, low‐density areas, high‐signal areas in white matter, MCA hyperdense vessel sign, collateral circulation score, perfusion imaging parameters (e.g., *T*_max_ > 10 seconds, cerebral blood flow < 30%), cerebral atrophy, ischemic lesion volume, reperfusion grade (mTICI), and midline shift. Additionally, we considered treatment information (e.g., ultraacute reperfusion therapy, intravenous thrombolysis, bridging therapy, endovascular treatment), complications (e.g., hyperlipidemia, alcohol abuse, active cancer, prestroke disability, respiratory infection), laboratory indicators (e.g., fasting blood glucose, glycated hemoglobin, C‐reactive protein, creatinine, neutrophil count, monocyte count, PCT, neuron‐specific enolase, blood calcium), and time variables (e.g., time of onset, hospital arrival time, reperfusion time). By integrating multidimensional data, the predictive power of the model was heightened, providing a more precise framework for assessing risk and making clinical decisions regarding poststroke cerebral edema.

Our study found that the predictive performance of ML models for long‐term cerebral edema did not show marked differences within 1 month, possibly because cerebral edema evolves. Empirical evidence suggests that cerebral edema typically peaks after a stroke and subsequently diminishes and gets absorbed. In the early stages, acute injury and inflammatory response significantly impact brain tissue, leading to edema and infiltration of inflammatory cells, potentially reaching a peak (Miao et al. 2020; Dhar 2020; Wu et al. 2018; Gunning et al. 2019). As inflammation decreases and neural tissue repairs, cerebral edema may be gradually alleviated or even completely resolved (Hua, Liu, and Wu 2023; Muscari et al. 2019; Miao et al. 2020; Wen et al. 2023; Pu et al. 2023). In summary, given the dynamic changes in cerebral edema, it is essential to consider specific predictive factors for evaluation and intervention strategies within different time windows (Miao et al. 2020).

Clinical evidence supports that radiomics methods provide substantial additional predictive power for traditional clinical features for forecasting cerebral edema. Wen et al. (2023) revealed the potential utility of texture features in identifying MCE after endovascular treatment in high‐risk patients, and the area under the curve (AUC) values obtained indicated high discriminative ability. L. Jiang, Zhang, et al. (2022) constructed predictive models based on radiomics features derived from MRI to predict cerebral edema after stroke and demonstrated excellent accuracy and reliability of these models. In our study, we initially searched for radiomics‐based studies, but ultimately, no articles met the inclusion criteria due to the absence of radiomics‐based methods specifically addressing cerebral edema in stroke patients. Therefore, our research lacked further exploration on radiomics.

In the process of improving predictive accuracy, in addition to efficient predictors, the selected model type is also an important factor affecting predictive results. Among predictive models in the included literature, logistic regression models were widely used. This preference may toile in the strong interpretability of logistic regression models, as their output can be viewed as probabilities (Gunning et al. 2019). However, some high‐performance methods, such as SVM and deep learning neural networks, lack interpretability (Gunning et al. 2019). This indicates that while these models may have higher predictive power, they may sacrifice some interpretability. In summary, model selection should consider both predictive performance and model interpretability, and the trade‐off between these two aspects is crucial for the development of clinical decision support systems.

While high‐performance methods such as SVM and deep learning neural networks demonstrate excellent predictive performance, trust issues may arise due to their “black‐box” nature, which may limit their application in clinical practice (Gunning et al. 2019). In clinical decision‐making, model interpretability is often considered a crucial factor, because assessment tools can be constructed based on simple and easily obtainable indicators, which strongly rely on interpretability. However, in image‐based deep learning applications, accuracy is more critical. Image processing is a complex process, and traditional ML methods usually show poor interpretability in extracting texture features. Therefore, in such cases, model accuracy is prioritized. In conclusion, when selecting models, it is essential to consider both predictive performance and model interpretability. Balancing these two aspects is crucial for clinical decision‐making. Future research should focus on developing models that leverage the predictive power of complex algorithms while providing clear interpretability for their predictions, thereby facilitating their practical application in clinical settings.

### 4.3 Strengths and Limitations

Regarding its strengths, this study provides seminal evidence for the field. However, there are also specific limitations. Despite a comprehensive literature search, the number of studies ultimately included in the analysis remains limited. This may affect the general applicability and interpretative power of the results. Second, the limited studies resulted in limited models included, which could potentially affect the interpretation of meta‐analysis results on the performance of different models. Third, the quantitative analysis of predictive factors was restricted to logistic regression models due to the absence of predictive factor weights for other models in the original studies. As a result, it was not feasible to accurately assess the contributions of these factors within their respective models.

## 5 Conclusions

Our systematic review has demonstrated that ML models can significantly predict cerebral edema following stroke. These models have exhibited high accuracy in validation cohorts, with a c‐index of 0.840, a sensitivity of 0.76, and a specificity of 0.87. These findings suggest that ML holds potential for improving clinical outcomes in stroke patients. For clinicians, these ML models can serve as powerful tools for early risk assessment, aiding in the rapid identification of patients at risk of developing severe cerebral edema. This may allow for more timely interventions, such as adjusting treatment plans or implementing preventive measures, which potentially improve patients' long‐term prognosis. Furthermore, as these models are further developed and validated, they can be integrated into electronic health record systems, thus providing real‐time support for clinical decision‐making.

However, given the limited number of studies included, future research should pay special attention to the following aspects: First, these models need to be further validated in larger and more diverse patient populations to ensure consistent predictive performance across different clinical settings. Second, user‐friendly interfaces need to be developed to enable clinicians to easily access and interpret the model outputs. Finally, the long‐term impact of these models needs to be investigated to assess their actual effect on improving patient outcomes and reducing healthcare costs.

Additionally, including studies from more diverse populations and geographical regions will enhance the generalizability and applicability of the models. Developing and clinically validating new predictors, particularly those based on biomarkers and advanced imaging techniques, will improve the predictive accuracy of the models. Exploring the practical application of ML models in different clinical settings, including their impact on patient management and treatment decisions, will further optimize the application of these models in predicting cerebral edema after stroke and provide stronger support for clinical practice.

In conclusion, while ML models have shown great potential in predicting cerebral edema after stroke, more efforts are needed before they can be widely applied in clinical practice, such as expanding sample sizes, increasing population diversity, and developing and clinically validating more effective predictors. By pursuing these specific research directions, future studies can further optimize the application of ML models in predicting cerebral edema after stroke and provide stronger support for clinical practice.

## Author Contributions

**Qi Deng**: writing–original draft, writing–review and editing, methodology, data curation, investigation, formal analysis. **Yu Yang**: writing–review and editing, formal analysis, investigation, data curation. **Hongyu Bai**: writing–review and editing, formal analysis, data curation, investigation. **Fei Li**: writing–review and editing, formal analysis, supervision. **Wenluo Zhang**: writing–review and editing, resources, visualization. **Rong He**: writing–review and editing, supervision, formal analysis. **Yuming Li**: writing–review and editing, supervision, visualization, project administration, data curation.

## Ethics Statement

The authors have nothing to report.

## Consent

The authors have nothing to report.

## Conflicts of Interest

The authors declare no conflicts of interest.

### Peer Review

The peer review history for this article is available at https://publons.com/publon/10.1002/brb3.70198.

## Supporting information




**Table S1** Literature search strategy.

**Table S2** Basic characteristics of enrolled literature.

**Table S3** Results of subgroup analysis based on different models and occurrence times.

## Data Availability

The original contributions presented in the study are included in the article, further inquiries can be directed to the corresponding author.
