# Supplementary material for: Predictive Value of Machine Learning Models for Cerebral Edema Risk in Stroke Patients: A Meta‐Analysis
Source: Brain Behav. 2025 Jan 8;15(1):e70198. doi: 10.1002/brb3.70198 (PMC11710891; doi:10.1002/brb3.70198)
Supplement: Supplementary file 1 — Table S1 Literature search strategy. [file BRB3-15-e70198-s001.docx]

# Supplementary Table 1 Literature search strategy

**1.Pubmed**

| Search number | Query | Results |
| --- | --- | --- |
| #1 | Strokes[MeSH Terms] | 178,859 |
| #2 | ((((((((((((((Strokes[Title/Abstract]) OR (Cerebrovascular Accident[Title/Abstract])) OR (Cerebrovascular Accidents[Title/Abstract])) OR (Cerebrovascular Apoplexy[Title/Abstract])) OR (Brain Vascular Accident[Title/Abstract])) OR (Brain Vascular Accidents[Title/Abstract])) OR (Apoplexy[Title/Abstract])) OR (Brain Infarctions[Title/Abstract])) OR (Brain Infarct[Title/Abstract])) OR (Brain Infarcts[Title/Abstract])) OR (Brain Infarction[Title/Abstract])) OR (Brain Venous Infarction[Title/Abstract])) OR (Brain Venous Infarctions[Title/Abstract])) OR (Venous Brain Infarction[Title/Abstract])) OR (Venous Brain Infarctions[Title/Abstract]) | 43,810 |
| #3 | machine learning[MeSH Terms] | 64,797 |
| #4 | (((((((((((((((((((((((((((machine learning[Title/Abstract]) OR (Transfer Learning[Title/Abstract])) OR (Deep learning[Title/Abstract])) OR (Ensemble Learning[Title/Abstract])) OR (artificial intelligence[Title/Abstract])) OR (random forest[Title/Abstract])) OR (neural network[Title/Abstract])) OR (neural networks[Title/Abstract])) OR (K-Nearest Neighbor[Title/Abstract])) OR (CNN[Title/Abstract])) OR (Support vector machine[Title/Abstract])) OR (SVM[Title/Abstract])) OR (Gradient Boosting Machine[Title/Abstract])) OR (Nomogram[Title/Abstract])) OR (XGBoost[Title/Abstract])) OR (Adaboost[Title/Abstract])) OR (Decision tree[Title/Abstract])) OR (ResNet-50[Title/Abstract])) OR (ResNet[Title/Abstract])) OR (Naive Bayesian[Title/Abstract])) OR (Multilayer perceptron[Title/Abstract])) OR (Bayesian network[Title/Abstract])) OR (Radiomics[Title/Abstract])) OR (Radiomic[Title/Abstract])) OR (Prediction model[Title/Abstract])) OR (Risk model[Title/Abstract])) OR (Risk factors[Title/Abstract])) OR (Predictors[Title/Abstract]) | 1,193,820 |
| #5 | Brain Edema[MeSH Terms] | 15,613 |
| #6 | ((((Brain Edema[Title/Abstract]) OR (Intracranial Edema[Title/Abstract])) OR (Brain Swelling[Title/Abstract])) OR (Brain Swellings[Title/Abstract])) OR (Cerebral Edema[Title/Abstract]) | 15,775 |
| #7 | (Strokes[MeSH Terms]) OR (((((((((((((((Strokes[Title/Abstract]) OR (Cerebrovascular Accident[Title/Abstract])) OR (Cerebrovascular Accidents[Title/Abstract])) OR (Cerebrovascular Apoplexy[Title/Abstract])) OR (Brain Vascular Accident[Title/Abstract])) OR (Brain Vascular Accidents[Title/Abstract])) OR (Apoplexy[Title/Abstract])) OR (Brain Infarctions[Title/Abstract])) OR (Brain Infarct[Title/Abstract])) OR (Brain Infarcts[Title/Abstract])) OR (Brain Infarction[Title/Abstract])) OR (Brain Venous Infarction[Title/Abstract])) OR (Brain Venous Infarctions[Title/Abstract])) OR (Venous Brain Infarction[Title/Abstract])) OR (Venous Brain Infarctions[Title/Abstract])) | 204,272 |
|  | (machine learning[MeSH Terms]) OR ((((((((((((((((((((((((((((machine learning[Title/Abstract]) OR (Transfer Learning[Title/Abstract])) OR (Deep learning[Title/Abstract])) OR (Ensemble Learning[Title/Abstract])) OR (artificial intelligence[Title/Abstract])) OR (random forest[Title/Abstract])) OR (neural network[Title/Abstract])) OR (neural networks[Title/Abstract])) OR (K-Nearest Neighbor[Title/Abstract])) OR (CNN[Title/Abstract])) OR (Support vector machine[Title/Abstract])) OR (SVM[Title/Abstract])) OR (Gradient Boosting Machine[Title/Abstract])) OR (Nomogram[Title/Abstract])) OR (XGBoost[Title/Abstract])) OR (Adaboost[Title/Abstract])) OR (Decision tree[Title/Abstract])) OR (ResNet-50[Title/Abstract])) OR (ResNet[Title/Abstract])) OR (Naive Bayesian[Title/Abstract])) OR (Multilayer perceptron[Title/Abstract])) OR (Bayesian network[Title/Abstract])) OR (Radiomics[Title/Abstract])) OR (Radiomic[Title/Abstract])) OR (Prediction model[Title/Abstract])) OR (Risk model[Title/Abstract])) OR (Risk factors[Title/Abstract])) OR (Predictors[Title/Abstract])) | 1,198,884 |
|  | (Brain Edema[MeSH Terms]) OR (((((Brain Edema[Title/Abstract]) OR (Intracranial Edema[Title/Abstract])) OR (Brain Swelling[Title/Abstract])) OR (Brain Swellings[Title/Abstract])) OR (Cerebral Edema[Title/Abstract])) | 24,217 |
|  | (((Strokes[MeSH Terms]) OR (((((((((((((((Strokes[Title/Abstract]) OR (Cerebrovascular Accident[Title/Abstract])) OR (Cerebrovascular Accidents[Title/Abstract])) OR (Cerebrovascular Apoplexy[Title/Abstract])) OR (Brain Vascular Accident[Title/Abstract])) OR (Brain Vascular Accidents[Title/Abstract])) OR (Apoplexy[Title/Abstract])) OR (Brain Infarctions[Title/Abstract])) OR (Brain Infarct[Title/Abstract])) OR (Brain Infarcts[Title/Abstract])) OR (Brain Infarction[Title/Abstract])) OR (Brain Venous Infarction[Title/Abstract])) OR (Brain Venous Infarctions[Title/Abstract])) OR (Venous Brain Infarction[Title/Abstract])) OR (Venous Brain Infarctions[Title/Abstract]))) AND ((machine learning[MeSH Terms]) OR ((((((((((((((((((((((((((((machine learning[Title/Abstract]) OR (Transfer Learning[Title/Abstract])) OR (Deep learning[Title/Abstract])) OR (Ensemble Learning[Title/Abstract])) OR (artificial intelligence[Title/Abstract])) OR (random forest[Title/Abstract])) OR (neural network[Title/Abstract])) OR (neural networks[Title/Abstract])) OR (K-Nearest Neighbor[Title/Abstract])) OR (CNN[Title/Abstract])) OR (Support vector machine[Title/Abstract])) OR (SVM[Title/Abstract])) OR (Gradient Boosting Machine[Title/Abstract])) OR (Nomogram[Title/Abstract])) OR (XGBoost[Title/Abstract])) OR (Adaboost[Title/Abstract])) OR (Decision tree[Title/Abstract])) OR (ResNet-50[Title/Abstract])) OR (ResNet[Title/Abstract])) OR (Naive Bayesian[Title/Abstract])) OR (Multilayer perceptron[Title/Abstract])) OR (Bayesian network[Title/Abstract])) OR (Radiomics[Title/Abstract])) OR (Radiomic[Title/Abstract])) OR (Prediction model[Title/Abstract])) OR (Risk model[Title/Abstract])) OR (Risk factors[Title/Abstract])) OR (Predictors[Title/Abstract])))) AND ((Brain Edema[MeSH Terms]) OR (((((Brain Edema[Title/Abstract]) OR (Intracranial Edema[Title/Abstract])) OR (Brain Swelling[Title/Abstract])) OR (Brain Swellings[Title/Abstract])) OR (Cerebral Edema[Title/Abstract]))) | 172 |

**2.Cochrane**

| Search number | Query | Results |
| --- | --- | --- |
| #1 | MeSH descriptor: [Stroke] explode all trees | 17122 |
| #2 | (Stroke):ti,ab,kw OR (Strokes):ti,ab,kw OR (Cerebrovascular Accident):ti,ab,kw OR (Cerebrovascular Accidents):ti,ab,kw OR (Cerebrovascular Apoplexy):ti,ab,kw | 75273 |
| #3 | (Brain Vascular Accident):ti,ab,kw OR (Brain Vascular Accidents):ti,ab,kw OR (Apoplexy):ti,ab,kw OR (Brain Infarctions):ti,ab,kw OR (Brain Infarct):ti,ab,kw | 2127 |
| #4 | (Brain Infarcts):ti,ab,kw OR (Brain Infarction):ti,ab,kw OR (Brain Venous Infarction):ti,ab,kw OR (Brain Venous Infarctions):ti,ab,kw OR (Venous Brain Infarction):ti,ab,kw | 5415 |
| #5 | (Venous Brain Infarctions):ti,ab,kw | 4 |
| #6 | MeSH descriptor: [Machine Learning] explode all trees | 951 |
| #7 | (machine learning):ti,ab,kw OR (Transfer Learning):ti,ab,kw OR (Deep learning):ti,ab,kw OR (Ensemble Learning):ti,ab,kw OR (artificial intelligence):ti,ab,kw | 6932 |
| #8 | (random forest):ti,ab,kw OR (neural network):ti,ab,kw OR (neural networks):ti,ab,kw OR (K-Nearest Neighbor):ti,ab,kw OR (CNN):ti,ab,kw | 4376 |
| #9 | (Support vector machine):ti,ab,kw OR (SVM):ti,ab,kw OR (Gradient Boosting Machine):ti,ab,kw OR (Nomogram):ti,ab,kw OR (XGBoost):ti,ab,kw | 2364 |
| #10 | (Adaboost):ti,ab,kw OR (Decision tree):ti,ab,kw OR (ResNet-50):ti,ab,kw OR (ResNet):ti,ab,kw OR (Naive Bayesian):ti,ab,kw | 1097 |
| #11 | (Multilayer perceptron):ti,ab,kw OR (Bayesian network):ti,ab,kw OR (Radiomics):ti,ab,kw OR (Radiomic):ti,ab,kw OR (Prediction model):ti,ab,kw | 6782 |
| #12 | (Risk model):ti,ab,kw OR (Risk factors):ti,ab,kw OR (Predictors):ti,ab,kw | 133893 |
| #13 | MeSH descriptor: [Brain Edema] explode all trees | 274 |
| #14 | (Brain Edema):ti,ab,kw OR (Intracranial Edema):ti,ab,kw OR (Brain Swelling):ti,ab,kw OR (Brain Swellings):ti,ab,kw OR (Cerebral Edema):ti,ab,kw | 2361 |
| #15 | #1 OR #2 OR #3 OR #4 OR #5 | 77591 |
| #16 | #6 OR #7 OR #8 OR #9 OR #10 OR #11 OR #12 | 146051 |
| #17 | #13 OR #14 | 2361 |
| #18 | #15 AND #16 AND #17 | 110 |

**3.Embase**

| Search number | Query | Results |
| --- | --- | --- |
| #1 | 'cerebrovascular accident'/exp | 451029 |
| #2 | stroke:ab,ti | 505655 |
| #3 | strokes:ab,ti | 46966 |
| #4 | 'cerebrovascular accident':ab,ti | 9119 |
| #5 | 'cerebrovascular accidents':ab,ti | 5239 |
| #6 | 'cerebrovascular apoplexy':ab,ti | 4 |
| #7 | 'brain vascular accident':ab,ti | 12 |
| #8 | 'brain vascular accidents':ab,ti | 4 |
| #9 | 'apoplexy':ab,ti | 5098 |
| #10 | 'brain infarctions':ab,ti | 556 |
| #11 | 'brain infarct':ab,ti | 1673 |
| #12 | 'brain infarcts':ab,ti | 1305 |
| #13 | 'brain infarction':ab,ti | 3676 |
| #14 | 'brain venous infarction':ab,ti | 1 |
| #15 | 'brain venous infarctions':ab,ti | 2 |
| #16 | 'venous brain infarction':ab,ti | 0 |
| #17 | 'venous brain infarctions':ab,ti | 1 |
| #18 | 'machine learning'/exp | 451444 |
| #19 | 'machine learning':ab,ti | 109482 |
| #20 | 'transfer of learning':ab,ti | 670 |
| #21 | 'deep learning':ab,ti | 59651 |
| #22 | 'ensemble learning':ab,ti | 2197 |
| #23 | 'artificial intelligence':ab,ti | 45748 |
| #24 | 'random forest':ab,ti | 27196 |
| #25 | 'neural network':ab,ti | 81355 |
| #26 | 'neural networks':ab,ti | 55584 |
| #27 | 'k-nearest neighbor':ab,ti | 4080 |
| #28 | 'cnn':ab,ti | 19893 |
| #29 | 'support vector machine':ab,ti | 25621 |
| #30 | 'svm':ab,ti | 21704 |
| #31 | 'gradient boosting machine':ab,ti | 1290 |
| #32 | 'nomogram':ab,ti | 26594 |
| #33 | 'xgboost':ab,ti | 4791 |
| #34 | 'adaboost':ab,ti | 1803 |
| #35 | 'adaboost':ab,ti | 1803 |
| #36 | 'decision tree':ab,ti | 18795 |
| #37 | 'resnet-50':ab,ti | 774 |
| #38 | 'resnet':ab,ti | 3276 |
| #39 | 'naive bayesian':ab,ti | 667 |
| #40 | 'multilayer perceptron':ab,ti | 3108 |
| #41 | 'bayesian network':ab,ti | 5318 |
| #42 | 'radiomics':ab,ti | 11673 |
| #43 | 'radiomic':ab,ti | 6422 |
| #44 | 'prediction model':ab,ti | 34582 |
| #45 | 'risk model':ab,ti | 11732 |
| #46 | 'risk factors':ab,ti | 872115 |
| #47 | 'predictors':ab,ti | 479185 |
| #48 | 'brain edema'/exp | 42755 |
| #49 | 'brain edema':ab,ti | 10436 |
| #50 | 'intracranial edema':ab,ti | 50 |
| #51 | 'brain swelling':ab,ti | 2372 |
| #52 | 'brain swellings':ab,ti | 6 |
| #53 | 'cerebral edema':ab,ti | 10769 |
| #54 | #1 OR #2 OR #3 OR #4 OR #5 OR #6 OR #7 OR #8 OR #9 OR #10 OR #11 OR #12 OR #13 OR #14 OR #15 OR #16 OR #17 | 653480 |
| #55 | #18 OR #19 OR #20 OR #21 OR #22 OR #23 OR #24 OR #25 OR #26 OR #27 OR #28 OR #29 OR #30 OR #31 OR #32 OR #33 OR #34 OR #35 OR #36 OR #37 OR #38 OR #39 OR #40 OR #41 OR #42 OR #43 OR #44 OR #45 OR #46 OR #47 | 1853897 |
| #56 | #48 OR #49 OR #50 OR #51 OR #52 OR #53 | 47924 |
| #57 | #54 AND #55 AND #56 | 614 |

**4.Web of science**

| Search number | Query | Results |
| --- | --- | --- |
| #1 | (((((((((((((((TS=(Stroke)) OR TS=(Strokes)) OR TS=(Cerebrovascular Accident)) OR TS=(Cerebrovascular Accidents)) OR TS=(Cerebrovascular Apoplexy)) OR TS=(Brain Vascular Accident)) OR TS=(Brain Vascular Accidents)) OR TS=(Apoplexy)) OR TS=(Brain Infarctions)) OR TS=(Brain Infarct)) OR TS=(Brain Infarcts)) OR TS=(Brain Infarction)) OR TS=(Brain Venous Infarction)) OR TS=(Brain Venous Infarctions)) OR TS=(Venous Brain Infarction)) OR TS=(Venous Brain Infarctions) |  |
| #2 | (((((((((((((((((((((((((((TS=(machine learning)) OR TS=(Transfer Learning)) OR TS=(Deep learning)) OR TS=(Ensemble Learning)) OR TS=(artificial intelligence)) OR TS=(random forest)) OR TS=(neural network)) OR TS=(neural networks)) OR TS=(K-Nearest Neighbor)) OR TS=(CNN)) OR TS=(Support vector machine)) OR TS=(SVM)) OR TS=(Gradient Boosting Machine)) OR TS=(Nomogram)) OR TS=(XGBoost)) OR TS=(Adaboost)) OR TS=(Decision tree)) OR TS=(ResNet-50)) OR TS=(ResNet)) OR TS=(Naive Bayesian)) OR TS=(Multilayer perceptron)) OR TS=(Bayesian network)) OR TS=(Radiomics)) OR TS=(Radiomic)) OR TS=(Prediction model)) OR TS=(Risk model)) OR TS=(Risk factors)) OR TS=(Predictors) |  |
| #3 | ((((TS=(Brain Edema)) OR TS=(Intracranial Edema)) OR TS=(Brain Swelling)) OR TS=(Brain Swellings)) OR TS=(Cerebral Edema) |  |
| #4 | #1 AND #2 AND #3 | 1071 |
