# Supplementary material for: Predictive Value of Machine Learning Models for Cerebral Edema Risk in Stroke Patients: A Meta‐Analysis
Source: Brain Behav. 2025 Jan 8;15(1):e70198. doi: 10.1002/brb3.70198 (PMC11710891; doi:10.1002/brb3.70198)
Supplement: Supplementary file 2 — Table S2 Basic characteristics of enrolled literature. [file BRB3-15-e70198-s002.docx]

**Supplementary Table 2** Basic Characteristics of Enrolled Literature

| **No** | **Author** | **Year** | **Country** | **Study Type** | **Source of Patients** | **Type of Stroke** | **Follow-up Duration** | **Number of Cerebral Edema Cases** | **Total Number of Cases** | **Total Number of Training Set Cases** | **Validation Set Generation Method** | **Number of Validation Set Cases** | **Type of Model Used** | **Modeling Variables** |
| --- | --- | --- | --- | --- | --- | --- | --- | --- | --- | --- | --- | --- | --- | --- |
| 1 | Zhang, Y. | 2018 | China | Cohort Study (Prospective) | Single-center | Acute Ischemic Stroke | 1 week | 25 | 93 | / | / | / | LR | Infarct Size in the Brain, Body Temperature, Neutrophil Count, Procalcitonin (PCT) Levels, Neuron-Specific Enolase (NSE) Levels |
| 2 | Zeng, W. X. | 2022 | China | Cohort Study (Prospective) | Single-center | Acute Ischemic Stroke | 90 days | 34 | 110 | 77 | Random Sampling | 33 | SVM/RF/XGBoost/KNN/GBM/LR | Baseline Demographic and Clinical Characteristics, Clinical Information Before and After Interventional Procedures, Brain Features on Non-Contrast Enhanced Computed Tomography (NCCT) Post-Mechanical Thrombectomy |
| 3 | Yoo, A. J. | 2013 | USA | Cohort Study (Prospective) | Single-center | Acute Ischemic Stroke | 90 days | 12 | 12 | / | / | / | LR | Age, Gender, Baseline NIHSS Score, Baseline DWI Lesion Volume, Time Factors, Follow-up DWI Lesion Volume, Receipt of IV tPA Treatment, NIHSS Score at Follow-up MRI, 90-Day NIHSS Score, 90-Day mRS Score, Outcomes Including Good Outcome (mRS 0-2), Favorable Outcome (mRS 0-4), Mortality Rate. |
| 4 | Xie, W. | 2023 | China | Cohort Study (Prospective) | Single-center | Acute Ischemic Stroke | 1 year | 120 | 312 | 165 | Random Sampling | 147 | LR | Pupils, Glasgow Coma Scale (GCS) Score, Acute Physiology and Chronic Health Evaluation (APACHE II) Score, National Institutes of Health Stroke Scale (NIHSS) Score, Alberta Stroke Program Early CT Score (ASPECTS), Collateral Score (CS), Admission Monocyte Count, White Blood Cell (WBC) Count, Glycated Hemoglobin (HbA1c) Level, History of Hypertension, History of Atrial Fibrillation. |
| 5 | Wen, X. H. | 2023 | China | Cohort Study (Retrospective) | Single-center | Acute Ischemic Stroke |  | 40 | 111 | 77 | / | 34 | LR | Gender, Age, History of Hypertension, Atrial Fibrillation, Hyperlipidemia, Diabetes, Alcohol Abuse, Smoking, Baseline NIHSS Score, Hyperdense MCA Sign, ASPECTS Score, Collateral Circulation Score, Occlusion Site, CBS, Ischemic Lesion Volume, Ischemic Core Volume, Intravenous tPA Use, Onset-to-Groin Puncture Time, Reperfusion Grade (mTICI), and Radiomics Markers. |
| 6 | Mohammadian Foroushani, H. | 2021 | USA | Cohort Study (Prospective) | Multi-center | Acute Ischemic Stroke And Subarachnoid Hemorrhage | / | / | 2246 | 2246 | / | / | / | / |
| 7 | Kumar, A. | 2022 | USA | Cohort Study (Retrospective) | Single-center | Acute Ischemic Stroke | 1 week | 53 | 160 | / | Internal Validation | / | / | / |
| 8 | Jiang, L. | 2022 | China | Cohort Study (Retrospective) | Single-center | Acute Ischemic Stroke | 36 hours | 90 | 272 | 212 | Random Sampling | 60 | RF/SVM/NB/KNN/AdaBoost/ANN | Modeling variables include MRI radiomics features from the infarct area and cerebrospinal fluid (CSF) characteristics. Specifically, MRI radiomics features provide quantitative information about the infarct zone, while CSF characteristics offer additional biomarker information. These features may specifically include shape, texture, and first-order features, with a total of 1,316 features extracted and incorporated into the modeling process. After selection and processing, these features are used to construct machine learning models to predict the occurrence of cerebral edema following acute ischemic stroke. |
| 9 | Jiang, L. | 2023 | China | Cohort Study (Retrospective) | Single-center | Acute Ischemic Stroke | 1 year | 46 | 1716 | 1256 | Random Sampling | 460 | Cox | Large Artery Occlusion, DWI ASPECTS Score, Previous Stroke mRS Score, Age, Bridging Therapy, Endovascular Treatment, Intravenous Thrombolysis, New Lesion, Hypertensive Encephalopathy, Hemorrhagic Transformation, Moderate to Severe Cerebral Infarction, Clinical-Radiological Composite Score (MRI-DRAGON Score), DWI Radiomics Features (Radscore). |
| 10 | Hoffman, H. | 2023 | USA | Cohort Study (Retrospective) | Single-center | Acute Ischemic Stroke | 90 days | 50 | 381 | 304 | Random Sampling | 77 | ANN/SVM/RF | Systolic Blood Pressure, Time Variables (such as Onset Time, Arrival Time, Reperfusion Time), Multiple Perfusion Imaging Parameters (such as Tmax > 10 seconds, Tmax > 6 seconds, Cerebral Blood Flow < 30%), Structured Clinical and Imaging Data, ASPECTS Score, NIHSS Score, Collateral Circulation Score, Reperfusion Status. |
| 11 | Fu, B. W. | 2020 | China | Cohort Study (Retrospective) | Single-center | Acute Ischemic Stroke | 24 hours | 39 | 116 | / | Random Sampling | 116 | RF/SVM/LR | The 16 variables include 13 imaging features and 3 clinical characteristics. The imaging features are extracted through histogram analysis, and the specific feature names may vary depending on the research methodology and data processing. The clinical characteristics include age, gender, and NIHSS score. |
| 12 | Foroushani, H. M. | 2022 | USA | Cohort Study (Retrospective) | Multi-center | Acute Ischemic Stroke | 24 hours | 20 | 598 | / | Random Sampling | 598 | LR/ANN | 1. Baseline Clinical and Imaging Variables 2. 24-hour NIHSS Score 3. ΔCSF 4. All Automated Imaging Variables, Including Hemispheric CSF Ratio 5. Midline Shift 6. Infarct Volume |
| 13 | Foroushani, H. M. | 2020 | USA | Cohort Study (Prospective) | Multi-center（3） | Cerebral Infarction | 24 hours | 20 | 361 | / | Random Sampling | 361 | LR | 1. Baseline Clinical Variables 2. Baseline Intracranial Reserve 3. ΔCSF Extracted After 24 Hours 4. Additional Quantitative Imaging Measurements |
| 14 | Dhar, R. | 2020 | USA | Cohort Study (Prospective) | Multi-center | Acute Ischemic Stroke | 7 days | 91 | 738 | / | Internal Validation | 738 | LR | Baseline NIHSS (National Institutes of Health Stroke Cabinet) Score, Baseline Cerebrospinal Fluid Volume, Glucose Levels, Time Factors (related to the rate of change in cerebrospinal fluid volume), Cerebral Edema Grades (CED grades), Age, Gender, Blood Pressure, tPA Treatment, TOAST Classification (Trial of ORG 10172 in Acute Stroke Treatment), Clinical Outcomes of Patients (such as progression of cerebral edema, post-stroke sequelae, etc.) |
| 15 | Dhar, R. | 2018 | USA | Cohort Study (Prospective) | Single-center | Cerebral Infarction | 24 hours | 32 | 155 | / | Random Sampling | 155 | GEE | Age, Time of Stroke Onset (T), Cerebral Edema Grades (CED Grade 3 vs. Grades 0, 1, 2) |
| 16 | Chen, Y. | 2016 | United States and Spain | Cohort Study (Retrospective) | Single-center | Acute Ischemic Stroke | 18 hours | 38 | 38 | 38 | Random Sampling+External Validation | 38 | RF | CSF (Cerebrospinal Fluid) Volume in CT Scans and ΔCSF (Change in CSF Volume Across Consecutive Scans) |
| 17 | Bustamante, A. | 2017 | Spain | Cohort Study (Prospective) | Multi-center | Cerebral Infarction | / | 383 | 12,227 | 12,227 | Random Sampling | 12,227 | RF/LR | Stroke Severity (NIHSS Score), Cerebral Edema, Age, Respiratory Infections, Symptomatic Intracranial Hemorrhage, Cardiac Complications, Admission to Stroke Unit, Dyslipidemia, Diabetes |
| 18 | Wu, S. M. | 2023 | China | Cohort Study (Prospective) | Multi-center | Acute Ischemic Stroke | 1 year | 232 | 2183 | 1627 | Random Sampling | 556 | LR | Age, Gender, Stroke Location, Stroke Type, Brain CT Imaging Features, Ultra-acute Reperfusion Treatment, Complications During Hospitalization. |
| 19 | Pu, M. J. | 2023 | China | Cohort Study (Prospective) | Single-center | Cerebral Infarction | 3 months | 48 | 307 | / | Random Sampling | 307 | LR | Time from Onset to Reperfusion, Systolic Blood Pressure, Admission Blood Glucose Levels, Baseline NIHSS Score, ASPECTS Score, Serum Creatinine Levels, etc. |
| 20 | Jiang, Q. M. | 2022 | China | Cohort Study (Retrospective) | Single-center | Cerebral Infarction | / | 72 | 329 | / | Random Sampling | / | LR | Hypertension, Previous Stroke, Consciousness Status on Admission, Calcium, Glucose, Intravenous Thrombolysis, Postoperative NIHSS Score, Stenting, Brain Atrophy, Hyperdense Middle Cerebral Artery (HMCA), Basal Cistern Effacement, Hypoattenuation Area, Postoperative Consciousness. |
| 21 | Du, M. | 2020 | China | Cohort Study (Retrospective) | Multi-center | Acute Ischemic Stroke | 5 days | 71 | 370 | 370 | Random Sampling | / | LR | Age, Baseline NIHSS Score, Collateral Circulation, Fasting Blood Glucose Level, and Recanalization Status. |
| 22 | Christoph Kurmann | 2022 | Switzerland | Cohort Study (Retrospective) | Single-center | Acute Ischemic Stroke | 3 months | / | 2,261 | 1,808 | Random Sampling | 453 | XGBoost | Stroke Severity, Age, Active Cancer, Pre-stroke Disability, Blood Glucose, C-Reactive Protein, Creatinine, White Matter Hyperintensities, Time from Onset to Admission. |
