# Supplementary material for: Predictive Value of Machine Learning Models for Cerebral Edema Risk in Stroke Patients: A Meta‐Analysis
Source: Brain Behav. 2025 Jan 8;15(1):e70198. doi: 10.1002/brb3.70198 (PMC11710891; doi:10.1002/brb3.70198)
Supplement: Supplementary file 3 — Table S3 Results of subgroup analysis based on different models and occurrence times. [file BRB3-15-e70198-s003.docx]

**Supplementary Table 3** Results of subgroup analysis based on different models and occurrence times

| **Subgroup** | **c-index** | | | | **Sensitivity and Specificity** | | |
| --- | --- | --- | --- | --- | --- | --- | --- |
|  | **n** | **Events** | **sampleSize** | **c-index(95%CI)** | **n** | **SEN (95%CI)** | **SPE (95%CI)** |
| Models |  |  |  |  |  |  |  |
| ANN | 4 | 63 | 768 | 0.86 (0.73 ~ 0.98) | 2 | 0.74-0.90 | 0.81-0.87 |
| SVM | 5 | 82 | 286 | 0.80 (0.76 ~ 0.84) | 4 | 0.70(0.58-0.80) | 0.85(0.76-0.91) |
| Adaboost | 1 | 22 | 60 | 0.75 (0.63 ~ 0.87) | 1 | 0.86 | 0.82 |
| GBM | 1 | 10 | 33 | 0.85 (0.68 ~ 1.00) | 1 | 0.50 | 0.87 |
| KNN | 2 | 32 | 93 | 0.81 (0.70 ~ 0.92) | 2 | 0.76-0.80 | 0.79-0.87 |
| LASSO | 3 | 84 | 363 | 0.92 (0.88 ~ 0.95) | 2 | 0.69-0.80 | 0.92-0.93 |
| LR | 9 | 279 | 1906 | 0.83 (0.78 ~ 0.89) | 6 | 0.75(0.69-0.81) | 0.86(0.78-0.91) |
| NB | 1 | 22 | 60 | 0.84 (0.74 ~ 0.94) | 1 | 0.87 | 0.81 |
| RF | 3 | 61 | 176 | 0.87 (0.74 ~ 1.00) | 2 | 0.85 | 0.86-0.95 |
| XGBoost | 1 | 10 | 33 | 0.87 (0.71 ~ 1.00) | 1 | 0.80 | 0.91 |
| **Time** | **c-index** | | | | **Sensitivity and Specificity** | | |
| ≤1month | 16 | 518 | 3526 | 0.84 (0.79 ~ 0.88) | 13 | 0.75(0.71-0.80) | 0.84(0.79-0.88) |
| ≥3 months | 12 | 241 | 1439 | 0.84 (0.80 ~ 0.88) | 7 | 0.79(0.68-0.86) | 0.91(0.86-0.94) |
| Overall | 30 | 665 | 3778 | 0.84 (0.81 ~ 0.87) | 22 | 0.76(0.72-0.79) | 0.87(0.83-0.90) |
